# Dengue virus serotype 4 in *Aedes aegypti* mosquitoes in Kenya

**DOI:** 10.1371/journal.pntd.0013856

**Published:** 2025-12-30

**Authors:** Ruut Joensuu, Viktor Olander, Moses Masika, Hanna Vauhkonen, C. Lorna Culverwell, Maija T. Suvanto, Lauri Kareinen, Kristian M. Forbes, Eili Huhtamo, Omu Anzala, Tarja Sironen, Olli Vapalahti, Teemu Smura, Essi M. Korhonen

**Affiliations:** 1 Department of Virology, Faculty of Medicine, University of Helsinki, Helsinki, Finland; 2 Department of Veterinary Biosciences, Faculty of Veterinary Medicine, University of Helsinki, Helsinki, Finland; 3 Department of Geosciences and Geography, Faculty of Science, University of Helsinki, Helsinki, Finland; 4 KAVI Institute of Clinical Research, Faculty of Health Sciences, University of Nairobi, Nairobi, Kenya; 5 Department of Medical Microbiology, Faculty of Health Sciences, University of Nairobi, Nairobi, Kenya; 6 Faculty of Biological and Environmental Sciences, Lammi Biological Station, University of Helsinki, Lammi, Finland; 7 Department of Biological Sciences, Fulbright College of Arts and Sciences, University of Arkansas, Fayetteville, Arkansas, Unites States of America; 8 HUS Diagnostic Center, Clinical Microbiology, University of Helsinki and Helsinki University Hospital, Helsinki, Finland; Colorado State University, UNITED STATES OF AMERICA

## Abstract

Dengue fever is one of the most globally significant arthropod-borne viral diseases. In 2024, more than 14 million cases and 10,000 deaths were reported across 92 tropical and subtropical countries. Dengue virus (DENV), endemic in Sub-Saharan Africa including Kenya, comprises four serotypes (DENV-1 to DENV-4). While DENV-1 to DENV-3 are widely distributed in the region, DENV-4 is considered rare. However, information on the distribution of DENV serotypes and the genetic diversity within African mosquito populations remains limited. To address this gap, 2,400 *Aedes aegypti,* the primary vector species of DENV, were collected from southeastern and coastal Kenya between 2016 and 2019 and subjected to viral analyses. Collected samples were screened for orthoflaviviruses using a nested pan-orthoflavi RT-PCR, and positive samples were Sanger sequenced. DENV-4 genotype I was detected in a pool of two female *Ae. aegypti* collected during a dengue outbreak in Mombasa in 2017, which was predominantly associated with DENV-2*.* The DENV-4 genome retrieved from this strain was similar to sequences of DENV-4 that have previously been reported from South India. We report the detection and genomic characterization of DENV-4 genotype I in Kenyan mosquito populations. These findings contribute to current knowledge of DENV serotype distribution in southeastern Africa and highlight the need for improved genomic surveillance to guide effective dengue prevention and control strategies.

## Author summary

Dengue fever is a mosquito-borne viral disease that affects millions of people each year, mostly in tropical and subtropical regions. The virus has four different serotypes, but in Africa, only three of them (DENV-1 to DENV-3) are commonly found. The fourth type, DENV-4, has been considered very rare on the continent, and little is known about where it occurs. In our study, we collected mosquitoes from southeastern and coastal Kenya between 2016 and 2019 to look for viruses they might be carrying. During this work, we found the presence of DENV-4 in mosquitoes collected in Mombasa during a 2017 outbreak. This is the first time DENV-4 has been identified in Kenyan mosquito populations. The phylogenetic analysis showed close relation to strains found in South India, suggesting that the virus may have been introduced from South Asia. Our findings expand current knowledge of distribution of dengue serotypes in Africa and highlight the importance of monitoring mosquito populations to identify different serotypes of dengue virus. Better surveillance can help improve outbreak preparedness and guide strategies to prevent and control dengue more effectively.

## Introduction

Dengue virus (DENV) is a positive-sense single-stranded RNA virus, classified under the genus *Orthoflavivirus* within the family *Flaviviridae* [1]*.* DENV consists of four distinct serotypes (DENV-1, -2, -3, and -4), of which DENV-1, -2, and -3 are the most prevalent worldwide, while DENV-4 is less frequently detected and has a more limited geographical distribution [2]. Each serotype is further classified into distinct genotypes; for example, DENV-4 includes five genotypes (I-V) [3]. Like in many tropical countries, DENV is endemic in Kenya, with several notable recent dengue outbreaks reported in 2017, 2019, 2022, and 2024 [4–6], which were characterized by the co-circulation of DENV-1, -2, and -3 [7–9]. While the presence of DENV-4 in Kenya has previously been confirmed through serological evidence, there is only limited genotypic information available on the circulating strains [7,8].

DENV infection in humans typically presents as an acute febrile disease characterized by biphasic fever, headache, myalgia, prostration, rash, lymphadenopathy, and leukopenia [6]. In some cases, the disease may progress to severe forms associated with vascular leakage, hemostatic abnormalities, and potentially life-threatening conditions including hypovolemic shock [10]. Following primary infection, individuals may acquire long-term immunity against the infecting serotype, but only short-term immunity against other serotypes. Secondary infection with a different serotype is considered a major risk factor for severe dengue, which can be life threatening [6]. Disease severity and clinical presentation can also vary by serotype and possibly genotype, although the association remains unclear. For example, DENV-4 has been associated with a higher frequency of respiratory and cutaneous symptoms compared with other serotypes [11]. While some studies suggest that DENV-4 is strongly associated with severe dengue, with even primary infections causing dengue hemorrhagic fever [12–14], others report DENV-4 being less frequently associated with severe disease than other serotypes [15–17].

Dengue virus is transmitted between humans by mosquito vectors. The primary vectors of DENV are *Aedes aegypti* and *Ae. albopictus*, with *Ae. aegypti* being widely distributed across Kenya [18]. This species has been implicated in multiple dengue and chikungunya outbreaks in the country, particularly in Mombasa and northeastern Kenya between 2013 and 2019 [6,19,20]. In Kenya, as in many other developing tropical countries, rapid urbanization—driven by increased trade, population growth, and human mobility—has created ecological conditions highly favorable for *Ae. aegypti* [21]. This trend is particularly pronounced in urban environments, where the abundance of artificial, non-biodegradable containers provides suitable breeding sites, often resulting in mosquito populations up to three times higher than those observed in rural areas [22]. Dengue serotype 4 is frequently involved in co-infections with other serotypes, particularly DENV-1 in populations of *Ae. aegypti* [23]. Notably, in such co-infections, DENV-4 has demonstrated a competitive replication advantage over DENV-1 [23].(Vazeille et al., 2016).

Genetic characterization of DENV strains in endemic regions is crucial, as genomic variation can influence viral fitness and clinical outcomes, as well as transmission patterns and geographic distribution [24,25]. Serotype 4 was first documented in the Philippines and Thailand in 1953 and has since spread to Americas and Africa [2]. In Africa, publicly available sequence data for DENV-4 remain scarce, with only 18 human-derived sequences reported to date: from Cameroon, Senegal, Nigeria, Angola, and Kenya [8,26–30]. In addition to the sequences from human samples, there are only eight mosquito-derived DENV-4 sequences publicly available from Africa; two from Cape Verde and six from Cameroon [30,31]. The limited availability of genomic data constrains our understanding of the evolution, emergence, and regional circulation of DENV-4. In this study, we investigated the presence of orthoflaviviruses in *Ae. aegypti* mosquitoes collected from southeastern and coastal Kenya between 2016 and 2019. Additionally, we aimed to determine the genetic origins of these orthoflavivirus(es) and to identify potential pathways of introduction.

## Methods

### Study area

**Fig 1:**
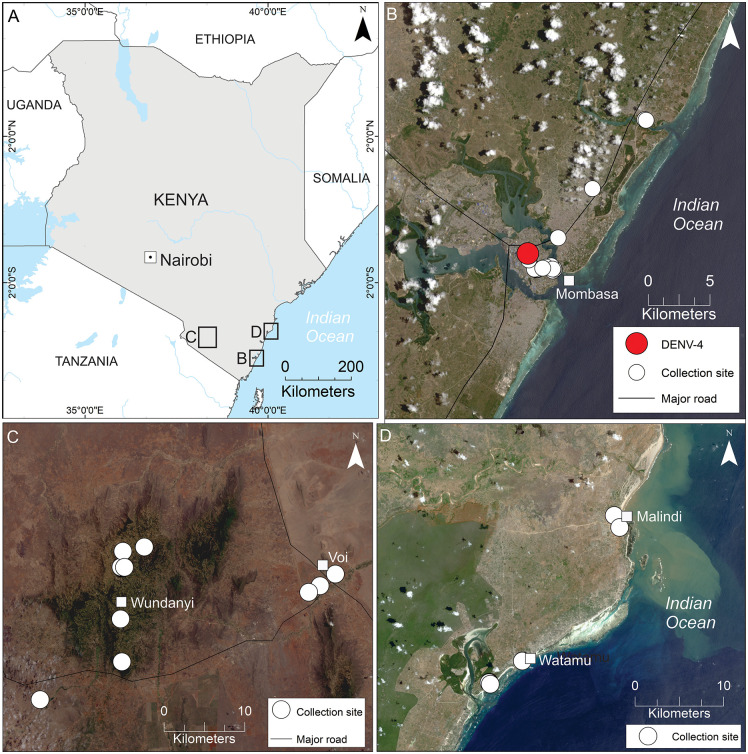
Map of Kenya in southeastern Africa (A), indicating mosquito collection sites in Mombasa City (B), the Taita Hills and surrounding lowlands in Taita–Taveta County (C), and Watamu and Malindi in Kilifi County (D), along with the location of virus positive DENV-4 mosquitoes. Maps were produced in ArcMap v. 10.8 (Esri, Redlands, CA, USA). Shapefiles for country boundaries and roads were obtained from Natural Earth [32], and Landsat 8–9 OLI/TIRS Level-1 scenes were downloaded from the USGS EarthExplorer [33].

Mosquito collections were conducted in three study areas in Kenya: Mombasa City in coastal Kenya, the Taita Hills and surrounding lowlands in Taita–Taveta County in rural southeastern Kenya, and the towns of Malindi and Watamu in Kilifi County in coastal Kenya (Fig 1). Mombasa City and Kilifi County have a super-humid, tropical climate with an average annual temperature of 27°C and 1,000 mm of rainfall [34]. Human population density varies significantly across the study regions, ranging from 5,966 inhabitants per km² in Mombasa City to 116 inhabitants per km² in Kilifi County. During the mosquito collections in Mombasa, a dengue outbreak with 1,200 reported human cases was ongoing [35–37]. The Taita Hills is a part of the Eastern Arc Mountains, which is recognized as one of the world’s 25 biodiversity hotspots, sustaining some of the richest concentrations of endemic flora and fauna [38]. It has a tropical highland climate, characterized by moderate temperatures and substantial rainfall, covering an area of approximately 850 km^2^, with altitudes ranging from 700 to 2,200 m above sea level [39]. The population density in the Taita Hills is approximately 534 inhabitants per km^2^ [40].

### Mosquito collections and initial processing

Mosquito collections were made in Kenya at several time points between 2016 and 2019, and across 68 sublocations, including Mombasa city (Fig 1B), the Taita Hills and surrounding lowlands in Taita–Taveta County (Fig 1C), and towns of Watamu and Malindi in Kilifi County (Fig 1D). Mosquitoes were collected both in urban areas (all study areas) and in forests and nature trails (Taita–Taveta and Kilifi Counties). Host seeking adult females were targeted using Prokopack aspirators (The John W. Hock Company, Gainesville, USA), and CDC Miniature Light Traps (The John W. Hock Company, Gainesville, USA) set up within 2 meters of ground level. The collection effort consisted of Prokopack aspirations lasting 15 minutes to one hour per collection event between 10am and 6pm, and light traps that were deployed overnight. Mosquitoes were individually examined under a stereomicroscope and morphologically identified using a combination of various identification keys [41–49]. Owing to the difficult nature of identifying African mosquito species, and fragmented/ incomplete keys, identifications were usually to genus level, but in the case of *Aedes* (*Stegomyia)*, they were identified to species level. Mosquitoes from other genera, including *Culex* and *Eretmapodites*, were also collected during sampling; however, they were excluded from the present analysis. Each collection was sorted individually, first by sex then to genus, and where possible to species, and in the case of larger collections up to 20 specimens of the same species were pooled prior to storage. Specimens were either stored in RNA-later (Thermo Scientific, Waltham, USA), or Virocult tubes (Medical Wire and Equipment, Corsham, UK). Any noticeably blood fed or gravid females were stored individually in 0.2 ml strip tubes with one of the two reagents and further pooled at a later point. While in Kenya, all specimens were maintained in a -20°C freezer until they were transported to the University of Helsinki, Finland, and stored at -80˚°C.

### Sample processing

Sterile sand, a tungsten bead, and either 900 µl (pools up to 9 individuals) or 1800 µl (pools up to 20 individuals) of Dulbecco’s PBS + 0.2% BSA to each tube. Each pool was then homogenized using the TissueLyser II (Qiagen, Germany) for 2 minutes at 30 rotations per second (rps). RNA extraction was performed from mosquito homogenate using 140 µl aliquots with the manual QIAamp Viral RNA Mini Kit and 200 µl aliquots with the QIAamp 96 Virus QIAcube HT Kit on the QIAcube HT or 200 µl aliquots with the MagMAX Pathogen Kit on KingFisher Duo automated purification system (Qiagen, Germany and Thermo Scientific, USA), respectively following the manufacturers’ instructions. The change in RNA extraction methods during the study period was necessitated by an update in laboratory automation systems, which required adopting protocols compatible with the available robotic platforms. Extracted RNA was treated with the DNA-free Kit (Thermo Scientific, Waltham USA) according to the manufacturer’s instructions to remove genomic insect-specific orthoflavivirus sequences. Subsequently, a two-step protocol for the detection of orthoflaviviruses (qRT-PCR followed by a nested PCR) was performed as described previously [50–51]. All nested PCR products were Sanger sequenced at the DNA Sequencing and Genomics Laboratory (BIDGEN) at the University of Helsinki for virus identification.

### Virus isolation

For virus isolation, 100 µl of the sample homogenate was mixed with 200 µl of sterile PBS and filtered through a 0.45 µm filter before being inoculated into a T25 flask containing C6/36 cells in a Biosafety Level 3 (BSL-3) laboratory. The cells were incubated at room temperature (RT) for one week before subculturing into a T75 flask. After subculturing, the cells were maintained for an additional week before harvesting. RNA was extracted from 100 µl of supernatant collected at different time points during the infections (days 1,3,6,7,10) using the Qiagen Viral RNA Kit (Qiagen, Germany). The extracted RNAs were screened for orthoflaviviruses using a two-step pan-orthoflavivirus RT-PCR as described previously [50,51]. Amplicons with a distinct band around ~200 base pairs were then selected for Sanger sequencing at BIDGEN.

### Whole-Genome Sequencing

Whole Genome Sequencing was performed for RNA from a DENV-4 positive mosquito pool using a DENV-4-specific tiled amplicon approach [52]. The obtained amplicons were sequenced with Oxford Nanopore Technologies (ONT) MinION using rapid barcoding RBK-004 sequencing kit, R9.4.1 flow cells and MinKNOW software v24.02.6 with fast basecall and default settings (ONT, United Kingdom).

The sequence reads were quality-filtered using Chopper [53] and mapped against the DENV-4 reference strain (MK858146) by Minimap2 [54]. Primer sequences were masked, and consensus sequence generated using iVar [55]. All DENV-4 complete coding sequences were downloaded from GenBank and aligned using MAFFT v7.505 [56]. The sequences were annotated to genotypes based on the lineage nomenclature proposed by [57], as implemented in Nextclade software [58]. Thereafter, the genotypes other than clade 4I_B.2 - into which the Kenyan strain was classified - were subsampled by first removing the sequences with >99% identity to each other followed by random subsampling within each genotype. The phylogenetic tree was constructed using the maximum-likelihood method implemented in IQ-TREE2 [59] and visualized using iTOL software [60].

## Results

### Mosquito data and RT-PCR screening

A total of 2,404 female *Ae. aegypti* were divided into 225 pools and then screened for orthoflaviviruses. Most of the mosquito pools were collected from Taita–Taveta County (N = 87) and Mombasa City (N = 84), with the lowest number obtained from Kilifi County (N = 54) (Table 1).

**Table 1:** Number of *Ae. aegypti* pools in each study area between 2016 and 2019, with Pan-orthoflavivirus PCR results and the corresponding average air temperature at each location. Average air temperatures were derived from ERA5 using ArcMap [61]. Taita–Taveta County includes the Taita Hills and surrounding lowlands, and Kilifi County includes Watamu and Malindi areas.

Collection year	Collection months	Collection location	Number of pools (Number of mosquitoes in pools)	Used RNA extraction Kit in each year	Pan-orthoflavivirus PCR result (Number of positives/ Total number of pools)	Average air temperature in the county (°C)
2016	Jan–March	Mombasa City	7 (N = 8)	MagMAX Pathogen Kit	5/7	28.5°C
		Taita–Taveta County	35 (N = 373)		18/35	26.3°C
		Kilifi County	0		0	28.3°C
		**Total**	**42 (N = 381)**		**23/42**	
2017	May	Mombasa City	32 (N = 243)	QIAamp Viral RNA Mini Kit	3/32	25.3°C
		Taita–Taveta County	3 (N = 31)		0/3	23.3°C
		Kilifi County	3 (N = 49)		1/3	25.8°C
		**Total**	**38 (N = 323)**		**4/38**	
			48 (N = 598)		8/48	25.5°C
2018	May	Mombasa City		QIAamp Viral RNA Mini Kit	3/6	23.2°C
		Taita–Taveta County	6 (N = 38)		5/51	26.1°C
		Kilifi County	51 (N = 464)		16/105	
		**Total**	**105 (N = 1100)**			
2019	Feb	Mombasa City	0	QIAamp 96 Virus QIAcube HT Kit	0	28.2°C
		Taita–Taveta County	40 (N = 600)		6/40	26.4°C
		Kilifi County	0		0	28.0°C
		**Total**	**40 (N = 600)**		**6/40**	

The initial Pan-orthoflavivirus PCR-screening detected 225 (N = 2,404 mosquitoes) positive pools of *Ae. aegypti*. After DNAse treatment, only 49 pools (N = 478 mosquitoes) remained positive (Table 1).

### Sequencing and phylogenetic analysis

**Fig 2:**
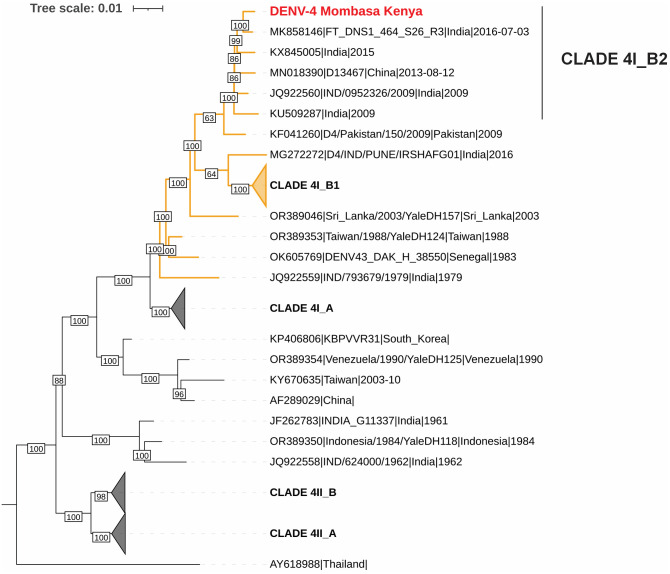
A phylogenetic tree constructed using partial coding sequence (77% coverage) of the DENV-4 strain from Mombasa and complete coding sequences of related reference sequences. The tree was inferred using maximum-likelihood method with GTR + F + R3 substitution model as determined by ModelFinder [62] implemented in IQTREE2 software. The new sequence is marked in red and clade 4I_B is shown in orange.

Of the 49 pan-orthoflavivirus pcr-products, we received 39 sequences and sequencing failed for 10 samples, possibly due to unspecific amplification and primer dimers (S1 Table). Among the 39 sequences, one amplicon, from a pool which consisted of two *Ae. aegypti* from Mombasa, was identified as DENV-4 using a NCBI BLAST search. The other 38 sequences were identified as insect-specific virus sequences. No evidence of DENV-1–3 was found. Whole genome sequencing with ONT MinION of the DENV-4 positive pool yielded a partial sequence with 77.2% breadth of coverage with a read depth exceeding 50x (mean depth: 751.45; median depth: 417. The sequence was deposited in GenBank under the following accession numbers: PX369881 (Submission ID: SUB15639668) (Fig 2).

The DENV-4 sequence from Mombasa clustered with Clade 4I_B.2 sequences from India and China. Its closest relative was a strain sequenced from a human serum sample collected in Bengaluru, India, in 2016, showing 99.3% nucleotide identity and 99.7% and amino acid identity [63].

### Virus isolation

Virus isolation was attempted from the mosquito pool which was found positive for DENV-4 in PCR screenings and subsequent sequencing. No cytopathic effect (CPE) was observed and DENV-4 was not detected from the RNA extracted from different time points.

## Discussion

Dengue is endemic in many African countries, including Kenya, and its burden has steadily increased over the past two decades. In Kenya, DENV-1, DENV-2, and DENV-3 have been the most frequently reported serotypes, and these have been primarily associated with documented outbreak events [5,37]. In contrast, DENV-4 has rarely been detected, and only in seropositive human samples [7,8]. Our finding of DENV-4, genotype I, in *Ae. aegypti* collected in Mombasa during the 2017 dengue outbreak represents molecular evidence of this serotype in mosquito populations in Kenya. This genotype of DENV-4 has not previously been reported from Kenya nor in East Africa.,

Globally and within Africa, DENV-4 remains relatively rarely detected serotype compared to other dengue virus serotypes [8,64]. It has been the least rapidly spreading serotype, although reports of its occurrence have been increasing in the last few decades [2]. In Africa, outside of Kenya, DENV-4 sequences from humans have only been reported from Angola, Cameroon, Nigeria and Senegal [65,66]. Additionally, mosquito-derived DENV-4 sequences have been documented from Western Africa from Cameroon and Cape Verde [30,31]. The rarity of DENV serotyping data in Africa may be influenced by limited resources and insufficient diagnostic infrastructure, which likely contribute to underdetection and underreporting of diseases [66]. The large dengue outbreak in 2017, which resulted in 1,117 cases and one death and coincided with the mosquito collections for this study in Mombasa, was reported to be associated with DENV-2 and DENV-1 based on patient samples [20,35,37,67]. However, our finding suggests that DENV-4 may have been silently co-circulating during this outbreak. Despite these constraints, the detection of DENV-4 genotype I in *Ae. aegypti* during the 2017 outbreak provides the first molecular evidence of this serotype in mosquito populations in Kenya, highlighting its potential role in local transmission and underscoring the need for enhanced arbovirus surveillance.

This study has some limitations that warrant consideration. DENV-4 was detected from a single pool of *Ae. aegypti* females, and we were unable to isolate the virus, likely due to the age of the sample and the use of storage media optimized for RNA preservation rather than virus viability. Consequently, viral infectivity could not be confirmed. This is also likely the reason why no DENV sequences were detected from RNA extracted at different timepoints of the isolation trials. In addition, the use of different RNA extraction kits over the study period may have introduced minor variability in PCR efficiency, although this is unlikely to have affected the overall interpretation of results. As only 77% of the DENV-4 genome was obtained through sequencing, there may be some limitations to the accuracy of the phylogenetic results. However, there are no sequence gaps in the E gene (positions 838–1498), which is the main gene used for DENV phylogeny. The missing sequence regions are also entirely in the regions coding for non-structural proteins (NS1, NS3, NS4A/B, NS5). Despite the lacking regions, the genome clustered with strain MK858146|FT_DNS1_464_S26_R3|India|2016-07-03 with maximal bootstrap support (100), improving the accuracy of the alignment.

Surveillance of vertebrate pathogens in mosquitoes is often challenging due to the high prevalence of insect-specific flaviviruses (ISFs), which can dominate sequencing results. In this study, we applied DNase treatment to improve the detection of vertebrate pathogens. Initially, PCR screening and Sanger sequencing yielded only ISF sequences. After DNase treatment of all positive mosquito pools, we successfully recovered a DENV-4 sequence from one pool. This demonstrates the added value of DNase treatment as a complementary approach for viral surveillance in mosquitoes. The phylogenetic analysis of circulating DENV strains is important for understanding transmission dynamics, geographical distribution, and the origins of the virus. While DENV infection is generally thought to provide lifelong immunity against symptomatic reinfection with the same serotype [68,69], it remains unclear whether this protection extends equally to all genotypes within that serotype [70]. Of the five genotypes of DENV-4, genotypes I and II are currently the most widely circulating in human populations [12,71,72]. Genotype I exhibits greater genetic diversity compared to other DENV-4 genotypes and some studies suggest that genotype I may be associated with more virulent strains, potentially contributing to increased disease severity and transmissibility [70,73]. Over the past century, genotype I has been reported across Southeast Asia, the Indian subcontinent, China, Brazil, and Australia, with phylogenetic evidence suggesting its likely origin in the Philippines, followed by spread to Thailand [65]. Furthermore, phylogenetic analysis provides insights into how DENV-4 may have spread from Asia into Africa and within Africa. The sequences most closely related to those in our study have been identified in Southern India [74]. Historically, DENV-3 genotype III was introduced from the Indian continent into East Africa [75], and more recently, DENV-2 of Indian origin was also detected in Kenya [76]. Given that Thailand, and particularly India, have served as key hubs for the regional and global dissemination of dengue viruses [64], it is plausible that also DENV-4 genotype I was introduced into Kenya and Mombasa from these regions. This scenario is further supported by the increasing trade and travel between Kenya, China, and India [77]. Particularly, Mombasa is often referred as “gateway to Africa” due to its status as the only major international port in Kenya, the largest commercial harbor in East Africa and a key logistics and infrastructure hub for the western Indian Ocean [78].

The global burden of dengue has increased significantly over the past two decades, with reported cases rising tenfold from 500,000 in 2000 to 5.2 million in 2019, a trend that is expected to continue, partly due to rising global temperatures. Consistent with these global patterns, Kenya experienced above-normal temperatures across most regions in 2023, reflecting a long-term warming trend accompanied by extreme weather events, including widespread flooding and drought in some areas [79]. Sustained increases in temperature are particularly concerning for dengue dynamics, as the optimal temperature for DENV transmission is around 29°C—higher than that of malaria, which peaks at around 25°C [80,81]. Consequently, higher air temperatures may favor dengue transmission over that of other vector-borne pathogens, such as malaria, which is nonetheless expected to become more prevalent in cooler highland regions of Africa, where it has historically been rare [6,82,83]. Globally, both the climatic suitability and the length of transmission season for dengue have increased substantially over recent decades [83]. The combined effects of climate change, rapid urbanization, and increased movement of people and goods in Africa are likely to further accelerate the spread of dengue and other arboviruses, highlighting the urgent need for strengthened surveillance and vector control strategies.

## Conclusions

The detection of DENV-4 genotype I in *Ae. aegypti* from Kenya provides novel insights into the diversity of circulating dengue virus serotypes in the region. Despite the constrains of the study, this finding strongly suggests that DENV-4 may have been silently co-circulating during the 2017 outbreak, likely at low prevalence and undetected by routine diagnostics. Such unnoticed circulation underscores the challenges of arbovirus surveillance, particularly when resources and diagnostic capacity are limited. This represents an important contribution to understanding DENV-4 distribution in Africa, where reports of this serotype remain scarce and sporadic. These results emphasize the need for sustained entomological surveillance and genomic monitoring, especially in regions with extensive international trade and human mobility, to detect changes in arbovirus circulation and support timely public health responses.

## Supporting information

S1 Table Closest significant alignment sequences for Sanger sequences of positive Pan-orthoflavivirus PCR amplicons. (XLSX)
